# An Australasian survey on the use of ChatGPT and other large language models in medical physics

**DOI:** 10.1007/s13246-025-01571-9

**Published:** 2025-05-20

**Authors:** Stanley A. Norris, Tomas Kron, Maeve Masterson, Mohamed K. Badawy

**Affiliations:** 1 Monash Health, Monash Imaging, Melbourne, Australia; 2 Peter MacCallum Cancer Centre, Department of Physical Sciences, Melbourne, Australia; 3 Sir Peter MacCallum Department of Oncology, University of Melbourne, Melbourne, Australia; 4 Department of Medical Imaging and Radiation Sciences, Monash University, Melbourne, Australia

**Keywords:** Medical physics, ChatGPT, Large language model, Generative artificial intelligence

## Abstract

This study surveyed medical physicists in Australia and New Zealand on their use of large language models (LLMs), particularly ChatGPT. There is currently no literature on the application of ChatGPT and other LLMs by medical physicists. This survey targeted a mixed group of professionals, including clinical medical physicists, registrars, students, and other specialised roles. It reveals that many respondents integrate LLM platforms into their work for a broad range of tasks. Most participants reported efficiency gains, although fewer perceived improvements in the overall quality of their work. Despite these benefits, substantial concerns remain regarding data security, patient confidentiality, and the lack of established guidelines or professional training for using these tools in a clinical context. Further, the potential for sudden changes in accessibility and pricing, which could disproportionately impact developing countries and under-resourced departments, implies that other vulnerabilities may exist. These findings suggest the need for the medical physics community to come together and debate the careful balance between exploiting LLM platforms and developing clear best practices that implement robust risk management strategies.

**Supplementary Information:**

The online version contains supplementary material available at 10.1007/s13246-025-01571-9.

## Introduction

**Fig. 1:**
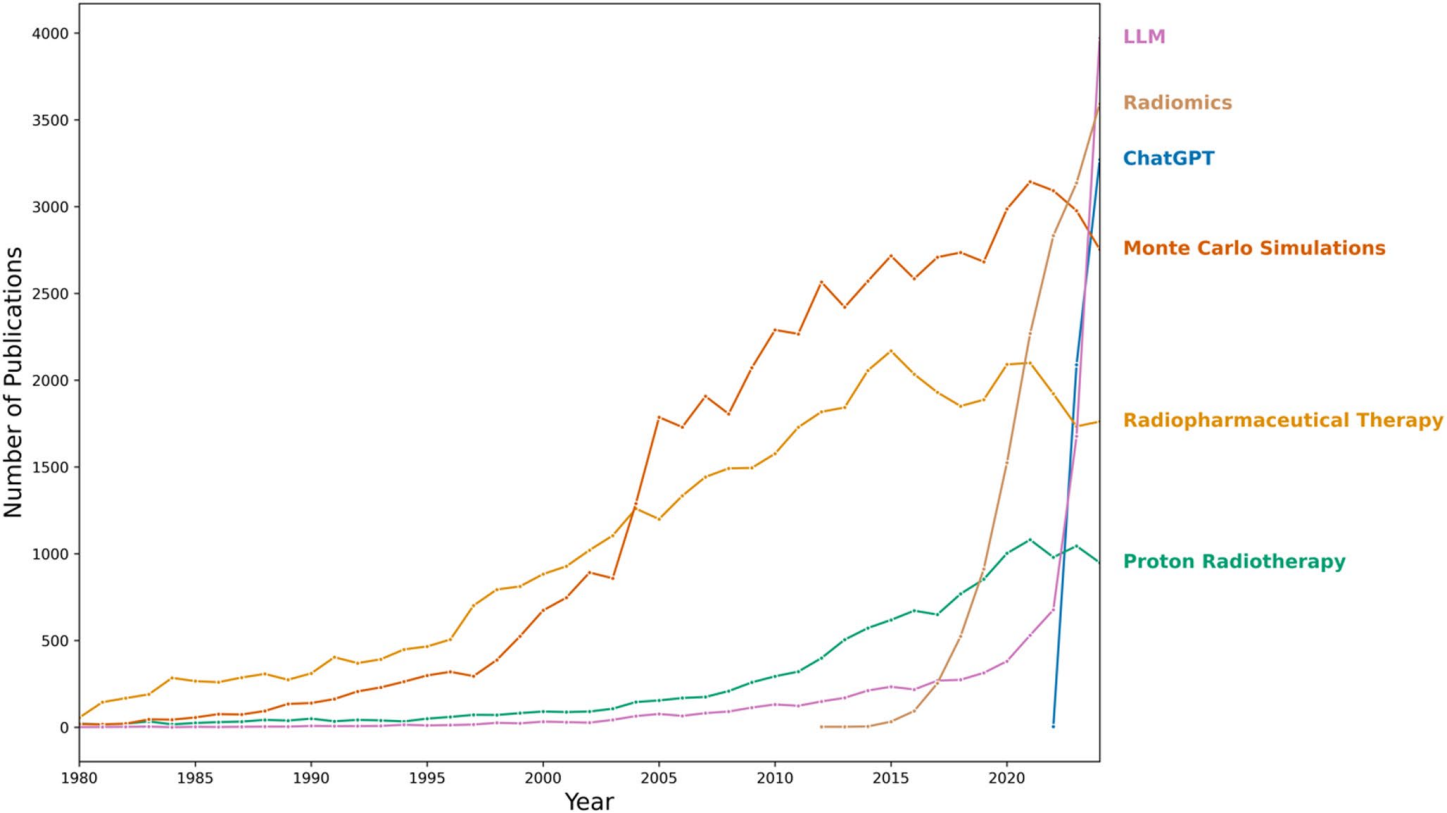
Plot of the number of new publications per year between 1980 and 2024, for different search terms in queries to the NIH’s National Library of Medicine PubMed database (accessed November 29th, 2024) [7]. Data points are omitted from the plot for years in which no studies were published on a particular term

The widespread availability of powerful large language models (LLMs), such as those offered by the ChatGPT platform (OpenAI, San Francisco, U.S.A.), requiring only a computer or mobile device with access to the internet, is set to have a lasting impact on society. Given their rapid adoption by general members of the public, as well as being the subject of a growing and broader research interest in Artificial Intelligence (AI), LLMs hold the promise to revolutionise the professional services industry [1–6]. The drive to develop AI models to improve or benefit the healthcare system is only part of much wider research efforts. A search of the PubMed National Institutes of Health (NIH) database under the term ‘Artificial Intelligence’ highlights its significance as a key area of research in medicine, with 45,083 new studies published in 2024 [7]. For a perspective against historical and current areas of medical physics, the number of publications in the PubMed database resulting for each of the search terms ‘Large Language Model’, ‘Radiomics’, ‘ChatGPT’, ‘Monte Carlo Simulations’, ‘Radiopharmaceutical Therapy’, and ‘Proton Radiotherapy’ plotted over time is shown in Fig. 1. The rapid increase in medical research related to the ChatGPT platform over two years has been remarkable. Undoubtedly, the high volume of ChatGPT research is partly due to its novelty and accessibility.

Many studies have been published assessing the performance of ChatGPT in tasks such as clinical decision-making [8–13], answering board-style examination questions [14, 15], generating, translating, and summarising diagnostic radiology reports [16–18], and streamlining patient communication [19]. Some of the biggest hurdles in translating AI products into clinical practice are those due to safety, privacy, governance, ethical, and regulatory issues. Nonetheless, private companies have developed AI and Machine Learning enabled devices that are currently being rolled out in different healthcare systems, following approval by regulatory bodies such as the Food and Drug Administration (USA), Therapeutic Goods Administration (Australia), and Medicines and Healthcare Products Regulatory Agency (UK).

Despite its rapid ascent in popular culture, ChatGPT—first released to the public in November 2022—is still in its infancy and seems to be met by the non-expert audience with curiosity and scepticism. Generative AI models can make unacceptable mistakes, such as hallucinations—instances where the model produces factually incorrect or fabricated information—and their deep complexity and black-box nature leave many perplexed as to how and why these tools work. Although some share the fear that they may lose their jobs to an AI model [20], it is essential to note the limitations of this technology. In most aspects of medicine, human verification is unlikely to be replaced entirely, because the consequences of an error could be catastrophic to the individual patient. However, if the principle of universal access is combined with a technology proven to increase efficiency, these models could drive much positive change for society.

A substantial number of studies have scrutinised the capabilities of ChatGPT and other LLMs in the context of diagnostic imaging, radiation oncology, and radiological protection physics [21–30]. Despite the value of AI being recognised by the medical physics community [31, 32], there is no literature on the role of ChatGPT in assisting and streamlining the day-to-day, non-specialised tasks of clinical, academic or research medical physicists. In response to this gap, we surveyed medical physicists to explore how they use the ChatGPT platform in professional contexts. To the authors’ knowledge, this study is the first to survey medical physicists on their use of the ChatGPT platform.

## Methods

### Recruitment details

The survey was distributed through the Australasian College of Physical Scientists and Engineers in Medicine (ACPSEM), which includes members from both Australia and New Zealand, as well as via publicly available mailing lists for medical physics professionals, including the Australian Medical Physics Register and the Victorian Department of Health’s list of approved medical physicists. The survey was also shared with academic medical physicists from 15 university departments across Australia and New Zealand.

To encourage participation in the survey, invited participants were entered in a draw to win a prize in the form of a medical physics T-shirt (While You Sleep, Melbourne, Australia) designed and hand-printed for this survey. Invited participants were given 18 days (31/01/2025-18/02/2025) to complete the survey before responses were collected and analysed for the study.

### Survey design and collection of data

The survey is designed to shed light on the patterns of ChatGPT usage by medical physicists of varying seniority levels and specialisations. Participants were prompted to enter their name and email address to collect the necessary information to identify and contact the prize winner. The first set of questions was defined so respondents could be grouped into experience levels and specialisations. These questions probed details about their role, years of experience, and specialisation(s). The second set of questions was designed to assess the patterns of use of ChatGPT (or an alternative LLM platform) in the work of these respondents. To ensure the survey is broadly applicable, we include a question to identify which LLM platform respondents might use as an alternative to ChatGPT, if applicable. These questions probed details about their experience using LLM platforms, the frequency at which they use them, whether they use the free or paid subscription service, what sort of tasks they use these tools for, whether they expect that their colleagues use one, whether they opt-in to allow the platform to use their content for model training, and how they believe these tools impact their work. An overview of the questions is provided in Table 1.

**Table 1:** Overview of survey structure and question types

	Question content	Response type	Questions
Identification	Name and email (for prize draw)	Short answer	2
Demographics	Role, years of experience, specialisation	Multiple choice	3
LLM Access & Awareness	Prior use of LLMs, specific platforms used, use by colleagues	Yes/No, multiple choice, short answer	3
Usage Frequency & Access	Frequency of use, paid version	Multiple choice, Yes/No	2
LLM Use Cases	Tasks categories where LLMs are used	Checkbox (multi-select)	1
LLM Consent Awareness	Opt-in for training data use	Yes/No/Not Sure	1
Perceived Impact	Beliefs about efficiency and quality improvements	Yes/No	2
Total			**14**

The survey was distributed using Microsoft Forms (Microsoft Corporation, Redmond, Washington, U.S.), a secure, web-based software platform. The Supplementary Information section provides the survey prompts, questions, and response options.

### Data analysis

The survey results were first exported as a Microsoft Excel (Microsoft Corporation, Redmond, Washington, U.S.) spreadsheet. Open text entries relating to alternative models were merged into a standard format to facilitate downstream analysis. In cases where open text consisted of feedback or reiterated the use of ChatGPT, these entries were omitted. Respondents who did not fit the survey criteria, such as those outside the medical physics profession (e.g., radiologists), were also omitted from the survey results. The analysis included summary statistics, that is, the number and proportion (in %) of respondents in each response category, which were performed and visualised using Python (v3.12.4). Following the pooling of results into each category of response, the data were visualised as bar charts. For ease of visualisation and analysis, several response categories were grouped. The roles Medical Physics Registrar, Medical Physics Trainee, and Master’s Student/Graduate were grouped as Medical Physicist in Training. Medical Physicist and Senior Medical Physicist were grouped as Qualified Medical Physicist, while Chief Medical Physicist, Deputy Chief Medical Physicist, Branch Head, and Principal Medical Physicist were grouped as Leadership Medical Physicist. Experience levels of ‘<3 years’ and ‘3–5 years’ were combined into a single ‘0–5 years’ group. Responses such as Radiobiology, Radiation Protection, and Software Engineering were grouped under ‘Other’ for the question regarding specialisations. For the question regarding roles, responses such as Research or Academic Medical Physicist, Medical Physics Software Engineer, Radiation Safety Officer, Contractor, and Retired Medical Physicist were grouped under ‘Other’.

## Results

**Fig. 2:**
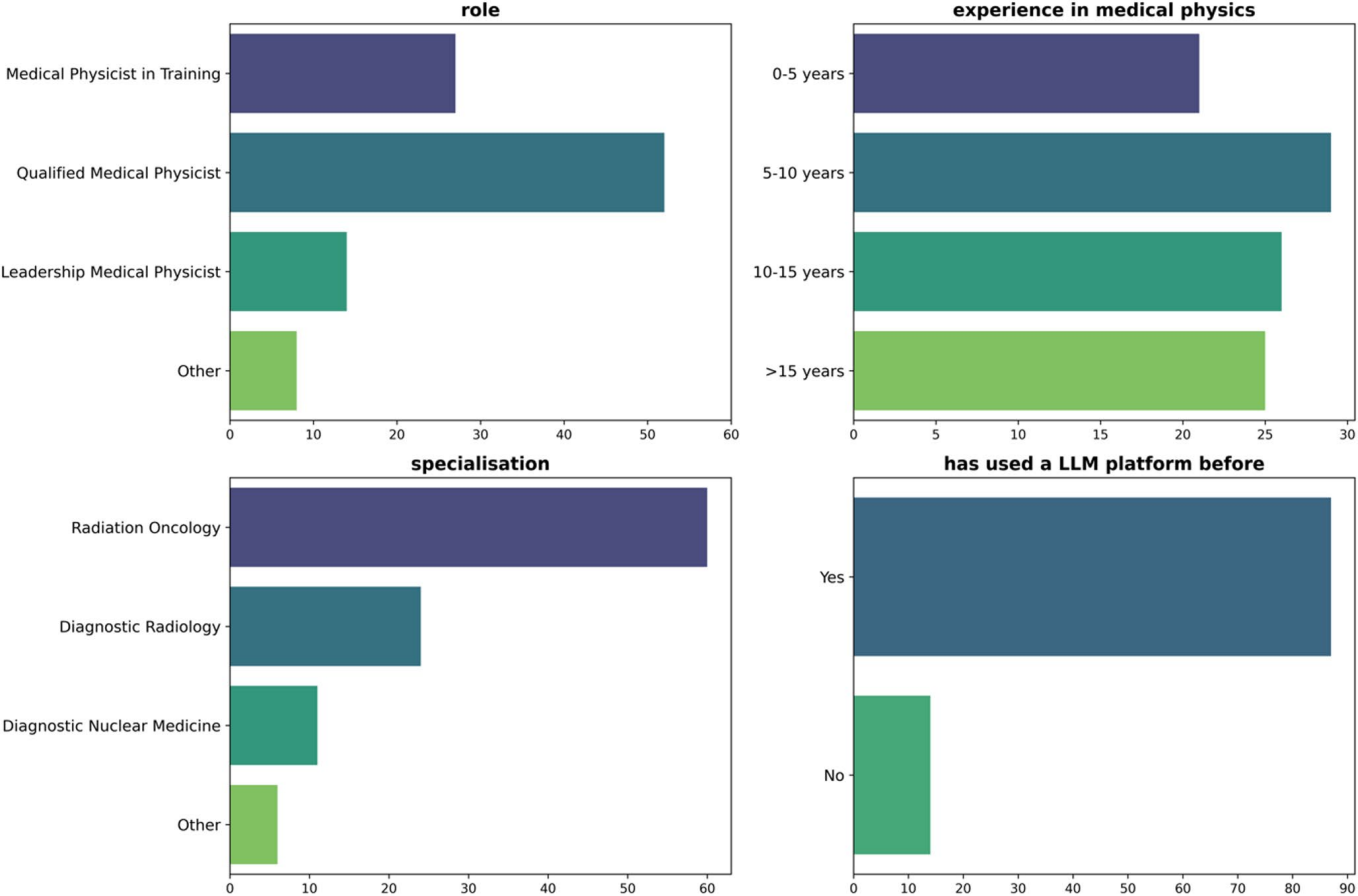
Responses to questions that probed respondent demographics and use of LLM platforms

A total of 101 individuals participated in the survey. The results summarising the distribution of roles, experience level, specialisation, and prior use of LLMs are shown in Fig. 2. Of the 101 respondents, 86% reported using a LLM platform at least once before, regardless of purpose or context. Responses between specialisations represented the workforce, with 59% of respondents in radiation oncology and 24% in diagnostic radiology.

**Fig. 3:**
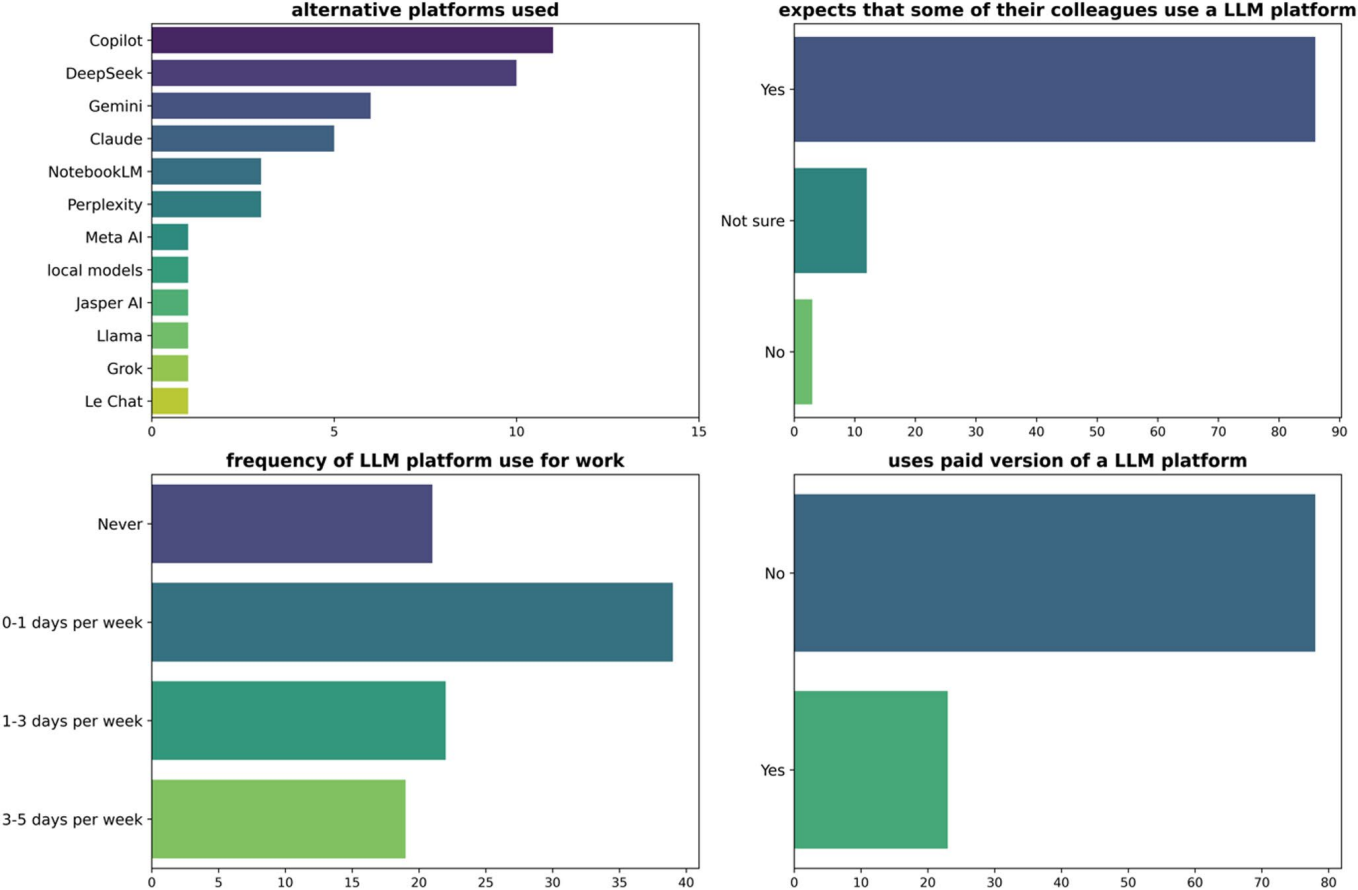
Responses to questions on the use of alternative platforms, frequency of use, perspective on the use of colleagues, and subscription to paid versions of LLM platforms

The results summarising the responses around alternative platforms, frequency of use, perspective on the use of LLMs by colleagues, and subscription status to the paid version of platforms are shown in Fig. 3. Respondents reported using many alternative platforms, at varying frequencies up to 3–5 days per week. The vast majority, 85% of respondents, expect some of their colleagues to use an LLM platform for their work in medical physics. However, much fewer (23%) respondents use the paid version of a LLM platform.

**Fig. 4:**
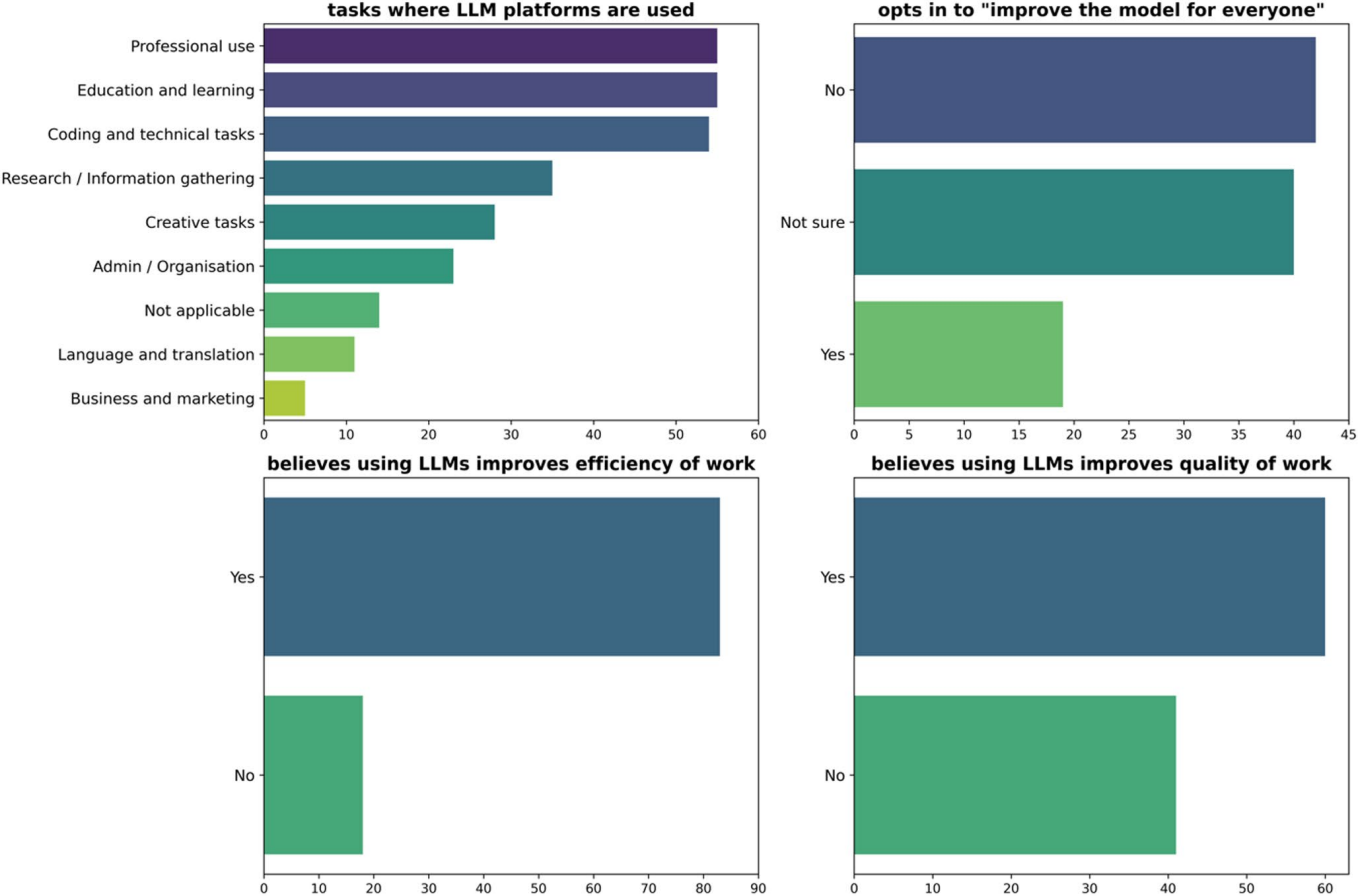
Responses to questions around tasks LLMs are used for, whether medical physicists opt-in to the platform using their data for model training, and their perspective around the impact these tools have on their work

The results concerning the tasks LLMs are used for, whether respondents opt in to the platform training on their data, and their perspective on the impact of these tools are shown in Fig. 4. Medical physicists use LLMs to cover a broad range of tasks. The most common responses included ‘professional use’, ‘education and learning’, and ‘coding and technical tasks’, reported by 54%, 54% and 53%, respectively. The survey responses to whether medical physicists opt in to “improve the model for everyone” were quite evenly mixed, with 41% answering ‘no’, 40% answering ‘not sure’, and 19% answering ‘yes’. In response to whether the participants believe that using LLM platforms improves their work efficiency, 82% chose ‘yes’. On the other hand, answering the question of whether participants believe that using LLM platforms improves the quality of their work, 59% chose ‘yes’.

**Fig. 5:**
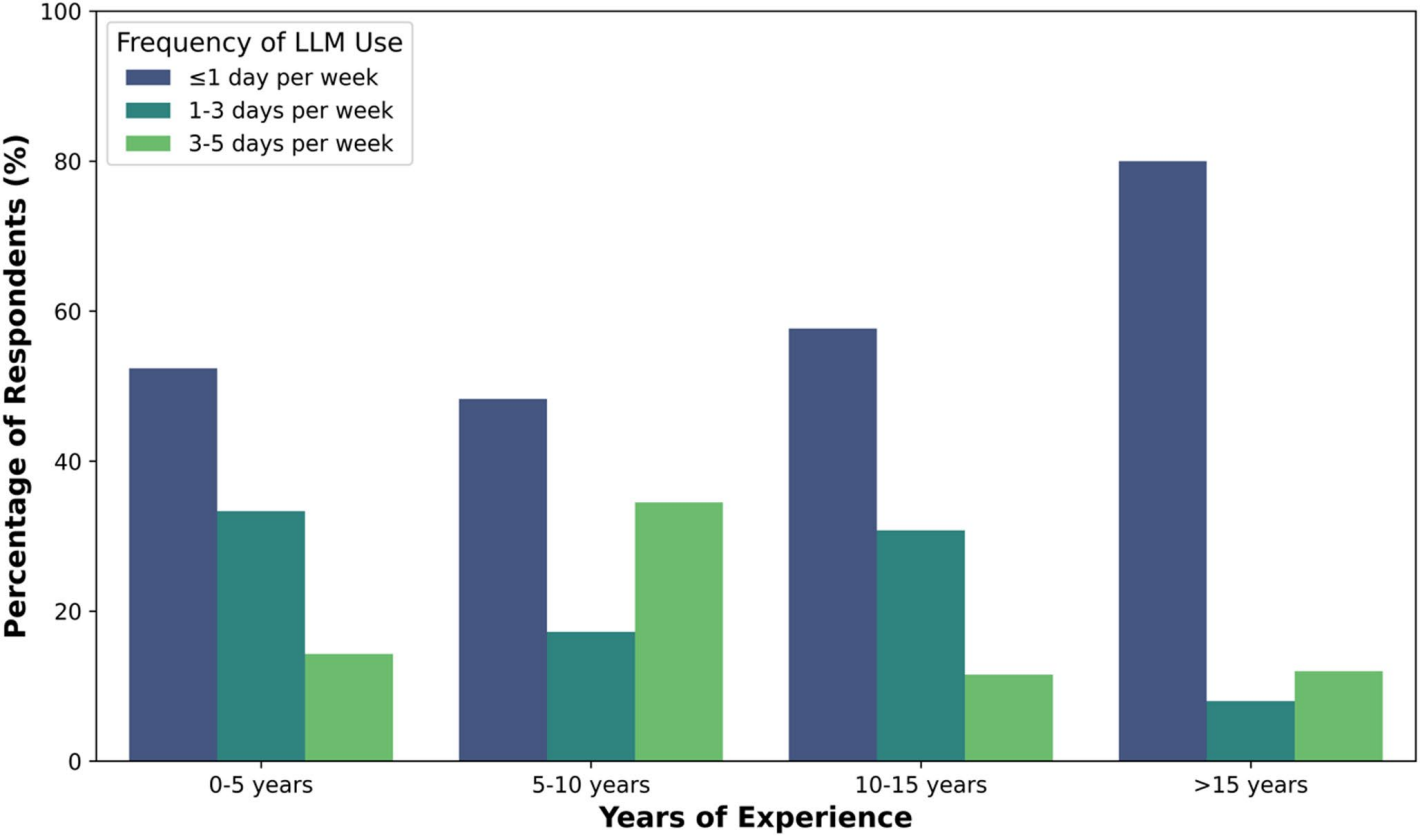
Frequency of LLM platform use (in %) among medical physicists, categorised by years of experience

To explore whether the frequency of use differs with experience, a grouped bar plot of the different frequencies (in %) of LLM platform use, grouped by years of experience, is shown in Fig. 5. Across all experience groups, most respondents use LLM platforms at a frequency of 1 day per week or less. Nonetheless, across all experience groups, many respondents regularly use LLM platforms multiple times per week. The group with 15 or more years of experience has the highest proportion of respondents who use LLM platforms less than 1 day per week (80%). In comparison, the 5–10 years of experience group has the highest proportion of respondents who use LLM platforms 3–5 times per week (35%). The experience group corresponding to 5 or fewer years has the highest proportion of respondents using LLM platforms 1–3 times per week (33%).

## Discussion

This survey captured various roles, experience levels, and specialisations. Although almost half the respondents were medical physicists or senior medical physicists, other respondents included medical physics registrars, students, research or academic medical physicists, principal and chief medical physicists, as well as other less common roles such as trainees, radiation safety officer, contractor, and software engineer. The demographics show that this survey captured many clinical medical physicists and other roles performed by the medical physics community. Most respondents had at least 5 years of experience in medical physics. Considering that years of experience do not include more general undergraduate studies (e.g., BSc in Physics), this survey captured a sample of relatively well-established professionals with substantial expertise within the field. Given that most of the medical physics workforce (~ 80%) consists of radiation oncology medical physicists [33], this proportion of survey respondents (60%) compared to diagnostic imaging medical physicists aligns with the distribution within the field. The survey also captured respondents in relatively niche areas of clinical medical physics, such as software engineering and radiobiology.

Most survey respondents have used an LLM platform before, and most respondents regularly use it in their work at frequencies up to 5 days per week. In contrast, fewer respondents (21%) report they ‘never’ use LLM platforms for their work in medical physics, and even fewer respondents (3%) do not expect their colleagues to use LLM platforms at work. Exploring the differences in the frequency of use across experience groups suggests that there may be a correlation between experience level and the lack of adoption of LLM platforms. However, the sample size is too small, and the number of respondents in each group is uneven, prohibiting a rigorous statistical evaluation. Moreover, it does not appear to be the case that the most junior medical physicists (with 0–5 years’ experience) use LLM platforms more frequently, given that the 5–10 years of experience group had the highest proportion of respondents using LLM platforms 3–5 times per week. Perhaps the relationship between the amount of time a medical physicist spends using a computer and their frequency of use of LLM platforms is more relevant. Despite a considerable number of medical physicists never using LLM platforms, it is clear that the community is acutely aware of the emergence of LLMs in the medical physics profession. However, 77% of medical physicists in this survey reported not using a paid version of these LLM platforms, indicating that they predominantly rely on free resources for their AI-driven tasks. This suggests that employers and healthcare organisations are either unaware of the utilisation of LLMs or do not consider it to be of enough value to pay for.

These findings indicate that professional medical physicists are familiar with or are actively adopting LLM platforms such as ChatGPT in their work. Although the main tasks LLMs are used for in medical physics are coding and technical tasks, education and learning, and professional use, LLMs are leveraged more broadly. The survey indicates this technology is also used for research and information gathering (e.g., literature summarisation), creative tasks (e.g., generating presentation content), administrative tasks (e.g., drafting emails), language and translation (e.g., simplifying technical content), and business and marketing (e.g., generating outreach materials). Most medical physicists responding to this survey believe that LLM platforms improve the efficiency of their work. Interestingly, fewer respondents believe that LLM platforms enhance the quality of their work. The survey question around whether medical physicists opt to “improve the model for everyone”, essentially using the platform to leverage user interactions to train models, is particularly important. A considerable proportion of respondents (39%) answered ‘not sure’, while the rest were evenly split between ‘yes’ and ‘no’. Currently, there is no consensus within the medical physics community on the best option. More research and education appear to be required.

For clinical use, there is a risk of creating a dependence on tools like ChatGPT, where sudden changes in accessibility and pricing structures could have negative consequences. A world health organisation (WHO) report on drug pricing stated that “Pharmaceutical companies set prices according to their commercial goals, with a focus on extracting the maximum amount that a buyer is willing to pay for a medicine” and found little evidence of a link between the costs of research and development and the price charged for the final product [34]. Given that an AI company could implement this strategy, we should at least be aware of our vulnerabilities. Sudden accessibility and pricing changes could disproportionately burden developing countries and under-resourced communities, exacerbating existing inequalities.

This survey-based research study captures the current landscape of LLM use in the medical physics profession. This study has found that many medical physicists in Australia and New Zealand actively leverage LLM platforms for a broad range of tasks in their day-to-day work. LLM platforms such as ChatGPT are already an asset to the medical physics community. This technology holds the most promise in automating the repetitive, administrative, and manual tasks that do not require responses grounded in a deep understanding of medical physics. Efficiency gains may be the most important benefit of using LLMs.

Given that we have shown LLM platforms have been adopted and are regularly used by 79% of respondents for work-related tasks in medical physics (i.e., those who did not select “Never” in response to the question on frequency of use), ethical, practical, and safety considerations remain subjects of ongoing debate. Careful thought should be given as to whether we should share our data to help improve LLM performance, weighing up potential benefits against privacy concerns. To ensure safe and effective use, strategies to minimise risk should be developed, particularly in tasks that could impact patients. Further, the trustworthiness of third-party AI companies remains an important concern due to a lack of transparency over data-handling policies. Addressing these issues through dedicated research and policy development will ensure that the medical physics community leverages this new technology responsibly and effectively.

A limitation of this study is that it is challenging to determine how many potential respondents were reached by the survey, as it was distributed via a mailing list and a post on the college’s website that may not have been seen by all medical physicists in Australia and New Zealand. Given that 199 email addresses were on the mailing list, this yields a response rate of ~ 50%. However, the estimated number of medical physicists in Australia and New Zealand would be closer to ~ 700 [33]. Additionally, some participants may have been on annual or sick leave or could not respond due to a high workload or the potential sensitivity of the information explored. These factors, combined with general difficulties inherent in recruiting external survey participants, limit our ability to provide a precise response rate and fully assess the sample’s representativeness. The survey was open briefly to represent a well-defined snapshot in time. In a rapidly developing field, this was considered to be important.

## Conclusion

This study suggests that LLM platforms such as ChatGPT have become an important part of the workflow of many medical physicists in Australia and New Zealand, enhancing efficiency by accelerating programming, text-based, and administrative tasks. Although there have been numerous research studies assessing the performance of LLM platforms in specific areas and for certain tasks, there is currently no data on the application of this technology by medical physics professionals. In other words, the questions around ‘what these tools are capable of’ have been asked, but the questions around ‘what medical physicists regularly use these tools for’ have not. This survey indicates a broad adoption across various roles, experience levels, and specialisations, reflecting a growing exploitation of these tools. However, this rapid adoption is accompanied by serious concerns regarding data security, patient confidentiality, and a lack of professional guidelines or training. Additionally, the potential for sudden changes in accessibility and pricing structures poses risks, particularly for developing countries and resource-constrained departments. These findings suggest the need for the medical physics community to evaluate the benefits and risks of LLM use critically and to establish educational guidelines and clear protocols and policies that safeguard sensitive information while enabling progress and equitable access to AI-driven technologies.

## Electronic Supplementary Material

Below is the link to the electronic supplementary material.

Supplementary Material
