# Supplementary material for: An Australasian survey on the use of ChatGPT and other large language models in medical physics
Source: Phys Eng Sci Med. 2025 May 20;48(3):1145–53. doi: 10.1007/s13246-025-01571-9 (PMC12511241; doi:10.1007/s13246-025-01571-9)
Supplement: Supplementary file 1 — Supplementary Material [file 13246_2025_1571_MOESM1_ESM.docx]

**Supplementary Information**

- ‘Enter your name’
- ‘Enter your email address’
- ‘Select which title describes your (main) role’
  - Master’s Student/Graduate
  - PhD student
  - Medical Physics Trainee
  - Medical Physics Registrar
  - Medical Physicist
  - Senior Medical Physicist
  - Principal Medical Physicist
  - Chief Medical Physicist
  - Radiation Safety Officer
  - Research/Academic Medical Physicist
  - Other
- ‘Select the number of years of experience you have in medical physics. The number of years of experience is defined as the total number of years you have been formally studying and/or working in medical physics.’
  - <3 years
  - 3-5 years
  - 5-10 years
  - 10-15 years
  - >15 years
- ‘Select your specialisation’
  - Diagnostic Radiology
  - Diagnostic Nuclear Medicine
  - Radiation Oncology
  - Radiation Protection
  - Health Physics
  - Other
- ‘Have you used ChatGPT (or an alternative platform such as Claude, Gemini, Llama, etc...) before?’
  - Yes
  - No
- ‘If you use an alternative LLM platform to ChatGPT, which one do you use?’
- ‘Do you expect that some of your colleagues use ChatGPT (or an alternative platform) for tasks relevant to their work in Medical Physics?’
  - Yes
  - No
  - Not sure
- ‘How frequently do you use ChatGPT (or an alternative platform) for tasks relevant to your work in Medical Physics?’
  - 0-1 days per week
  - 1-3 days per week
  - 3-5 days per week
  - Never
- ‘Do you use the paid version of the ChatGPT (or an alternative platform)?’
  - Yes
  - No
- ‘Select all tasks in which you use or have used ChatGPT (or an alternative platform)’
  - Education and learning
  - Professional use (drafting emails, generating reports, writing resumes, etc...)
  - Creative writing and content creation
  - Research and information gathering
  - Coding and technical tasks
  - Administrative and organisational tasks
  - Language and translation
  - Business and marketing
  - Not applicable
- ‘Do you opt in to “improve the model for everyone”, essentially allowing your content to be used to train the platform’s models?’
  - Yes
  - No
  - Not sure
- ‘Do you believe that using ChatGPT (or an alternative platform) improves your efficiency at completing certain tasks?’
  - Yes
  - No
- ‘Do you believe that using ChatGPT (or an alternative platform) improves the quality of your work?’
  - Yes
  - No
